# Blood Pressure Targets for Out-of-Hospital Cardiac Arrest: A Systematic Review and Meta-Analysis

**DOI:** 10.3390/jcm12134497

**Published:** 2023-07-05

**Authors:** Shir Lynn Lim, Christopher Jer Wei Low, Ryan Ruiyang Ling, Rehena Sultana, Victoria Yang, Marcus E. H. Ong, Yew Woon Chia, Vijay Kumar Sharma, Kollengode Ramanathan

**Affiliations:** 1 Department of Cardiology, National University Heart Centre Singapore, Singapore 119074, Singapore; 2 Yong Loo Lin School of Medicine, National University of Singapore, Singapore 117597, Singapore; 3 Pre-Hospital Emergency Research Center, Duke-NUS Medical School, Singapore 169857, Singapore; 4 Centre for Quantitative Medicine, Duke-NUS Medical School, Singapore 169857, Singapore; 5 Imperial College Healthcare NHS Trust, London W12 OHS, UK; 6 Department of Emergency Medicine, Singapore General Hospital, Singapore 169608, Singapore; 7 Health Services and Systems Research, Duke-NUS Medical School, Singapore 169857, Singapore; 8 Department of Cardiology, Tan Tock Seng Hospital, Singapore 308433, Singapore; 9 Lee Kong Chian School of Medicine, Nanyang Technological University, Singapore 639798, Singapore; 10 Division of Neurology, National University Health System, Singapore 119074, Singapore; 11 Cardiothoracic Intensive Care Unit, National University Heart Centre Singapore, Singapore 119074, Singapore

**Keywords:** out-of-hospital cardiac arrest, blood pressure, haemodynamics, neurologic deficits, meta-analysis

## Abstract

Background: With ideal mean arterial pressure (MAP) targets in resuscitated out-of-hospital cardiac arrest (OHCA) patients unknown, we performed a meta-analysis of randomised controlled trials (RCTs) to compare the effects of higher versus lower MAP targets. Methods: We searched four databases until 1 May 2023 for RCTs reporting the effects of higher MAP targets (>70 mmHg) in resuscitated OHCA patients and conducted random-effects meta-analyses. The primary outcome was mortality while secondary outcomes were neurological evaluations, arrhythmias, acute kidney injury, and durations of mechanical ventilation and ICU stay. We conducted inverse-variance weighted strata-level meta-regression against a proportion of non-survivors to assess differences between reported MAPs. We also conducted a trial sequential analysis of RCTs. Results: Four RCTs were included. Higher MAP was not associated with reduced mortality (OR: 1.09, 95%-CI: 0.84 to 1.42, *p* = 0.51), or improved neurological outcomes (OR: 0.99, 95%-CI: 0.77 to 1.27, *p* = 0.92). Such findings were consistent despite additional sensitivity analyses. Our robust variance strata-level meta-regression revealed no significant associations between mean MAP and the proportion of non-survivors (B: 0.029, 95%-CI: −0.023 to 0.081, *p* = 0.162), and trial sequential analysis revealed no meaningful survival benefit for higher MAPs. Conclusions: A higher MAP target was not significantly associated with improved mortality and neurological outcomes in resuscitated OHCA patients.

## 1. Introduction

Out-of-hospital cardiac arrest (OHCA) portends dismal outcomes [1]. Only 40% of those admitted to hospital following OHCA survive to hospital discharge; even fewer have neurologically intact survival [2,3]. Narrowing the gap between survival to hospital admission and discharge would save many lives, underscoring the urgent need to improve post-arrest care. Brain injury accounts for two-thirds of deaths in patients resuscitated from OHCA [2], yet therapies reducing the impact of global cerebral ischaemia following cardiac arrest remain unclear. The mechanisms of brain injury following OHCA, and subsequent resuscitation, are complex. Many pathways are activated between hours and days after the return of spontaneous circulation (ROSC), providing a potential treatment window for neuroprotection after ROSC [4,5,6,7]. 

One key element of neuroprotection is optimising cerebral blood flow, which is determined by mean arterial pressure (MAP) and cerebrovascular resistance. There is limited evidence on haemodynamic management after resuscitation, and the optimal MAP is unclear. Based on guidelines in sepsis [8], current guidelines recommend a MAP ≥ 65 mmHg [9], which may provide inadequate cerebral oxygenation during the critical initial 6–12 h of intensive care unit (ICU) stay (in the delayed hypoperfusion phase) [10,11]. Landmark clinical trials have not yet substantiated these guidelines. A prior systematic review suggested improved clinical outcomes with higher MAP targets (MAP 65–90 mmHg, SBP of 90–100 mmHg) [12]. However, this review was limited by observational data and significant variations in haemodynamic thresholds among studies, thus precluding a meta-analysis. Subsequent randomised controlled trials (RCTs) failed to detect differences in outcomes between different MAP targets, and no prior meta-analysis of the trials has been conducted [13,14,15].

The conflicting findings in the prevailing evidence highlight the need for an updated review of the literature. Accordingly, we report this systematic review of RCTs to compare the effects of higher versus lower MAP targets in the early post-resuscitation phase on survival and neurological outcomes, by adopting a clearly defined MAP target. 

## 2. Methods

### 2.1. Search Strategy and Selection Criteria

We registered this study on PROSPERO (CRD42022319242) and conducted it in adherence with the Preferred Reporting Items for Systematic Reviews and Meta-analyses Statement (Supplementary Table S1) [16]. We collaborated with a medical information specialist and searched MEDLINE via Pubmed, Embase, Cochrane, and Scopus using the keywords “out-of-hospital cardiac arrest” and “blood pressure” from origin through 1 May 2023 (Supplementary Table S2). We reviewed the reference lists of included studies and review articles. We included studies reporting on the effects of MAP after being resuscitated from OHCA. We excluded animal studies, correspondences, reviews, and non-English publications. In cases where the same patient data were reported in two or more studies (i.e., overlapping data), we included the largest study. Furthermore, prior RCTs had used varying thresholds for higher MAPs (between 72 mmHg and 85–100 mmHg) [13,15]. In order to comprehensively evaluate these available RCTs, we prespecified 70 mmHg as the threshold value in our meta-analysis. To be included, studies must report one group with a MAP ≤ 70 mmHg, and another with >70 mmHg. For studies which report a range of MAP targets (e.g., 65–75 mmHg), we used the median value (70 mmHg) to represent the MAP of the group. If any were available, studies with more than two study groups were also included. 

### 2.2. Data Collection and Risk of Bias Assessment

We collected data using a prespecified data extraction form (Supplementary Table S3). We rated the intra-study risk of bias using the Cochrane Risk-of-Bias (RoB) Tool 2.0 for RCTs [17]. We assessed the overall certainty of evidence using the Grading of Recommendations, Assessment, Development and Evaluations (GRADE) approach [18]. The screening of studies, data collection, and risk of bias assessment were conducted independently in duplicate by CJWL, VY, and RRL; conflicts were resolved by consensus, KR, or SLL.

### 2.3. Data Synthesis

The primary outcome was pooled mortality while secondary outcomes included favourable neurological recovery, defined by a Cerebral Performance Category (CPC) score of 1–2 or modified Rankin score (mRS) of 0–2 [19,20], or neuron-specific enolase levels at 48 h, which have been recommended as part of multimodal neuromonitoring in post-resuscitation care [21]. We also analysed incidences of arrhythmias and acute kidney injury (AKI) as well as days of intensive care unit (ICU) stay and mechanical ventilation as secondary outcomes. Other complications were not consistently reported; we report these outcomes qualitatively. Statistical analyses were performed using R4.0.5 using the *meta*, *robumeta*, and *metafor* packages. We did random-effects meta-analyses (DerSimonian and Laird) based on the logit transformation and computed 95% confidence intervals (CIs) using the Clopper–Pearson method [22,23,24,25]. Dichotomous outcomes are presented as pooled odds ratios (OR), and continuous outcomes as pooled mean differences, each with their corresponding 95% CIs. We also pooled hazard ratios (HRs), where reported. 

We conducted three separate sensitivity analyses for this study. First, in view of one of the studies reporting a MAP range of 65–75 mmHg, of which we took the median 70 mmHg to be the target MAP of the group, we conducted a sensitivity analysis excluding this study. Secondly, to account for the low number of RCTs on this topic, investigate differences between RCTs and observational studies, and improve the precision of our estimates, we further conducted a sensitivity analysis including only selected observational studies meeting our prespecified MAP criteria that had 70 mmHg as a MAP threshold. Finally, we further explored the possibility of a dose–response relationship of MAP targets between 70–80 mmHg and above 80 mmHg. We assessed for publication bias by visually inspecting funnel plots and Egger’s test. 

We conducted a prespecified subgroup analysis based on the number of centres involved and the duration of follow up (discharge to 30 days or 90 to 180 days). Where not presented, we derived the means and standard deviations from the data presented in each study in accordance with Wan et al. [26]. We assessed the inter-study heterogeneity using GRADE, using the I^2^ values, tau-squared values, and *p*-value from the Cochran Q test, as well as qualitatively by visualising forest plots [27]. A *p*-value of <0.05 was considered significant in our analysis. 

Furthermore, we used inverse-variance weighted strata-level meta-regression to derive a summary effect estimate of the pooled proportion of non-survivors at various MAPs. We collected MAP values for each group amongst any studies that reported mean MAP values, and derived means from any studies reporting median MAP values. We estimated the standard errors using robust variance estimates by clustering the pooled proportions around each unique study identifier to account for intra-study correlation. We regarded the study identifier as a random-effects variable and incorporated the reported mean MAPs as an independent variable, which was modelled as a continuous variable. 

Finally, for all outcomes, we conducted a trial sequential analysis (TSA) using TSA v0.9.5.10 (www.ctu.dk/tsa, accessed on 10 May 2023), assessing efficacy based on the O’Brien–Fleming alpha-spending function, and futility based on the beta-spending function. TSA combines cumulative meta-analysis with a sample size calculation to evaluate a cumulative pooled effect after an additional trial is included based on the information size thus obtained in a similar fashion to group sequential monitoring boundaries in RCTs during interim analyses [28]. We estimated the required information size (RIS) and cumulative Z-scores using the relative risk reduction and baseline estimates of the low MAP group based on the results of our meta-analysis. We estimated the variance of the pooled estimate and heterogeneity using the TSA software. We assumed a type I error of 5% and a power of 80%. A *p*-value of <0.05 was defined as statistically significant for our analyses. We performed all statistical analyses using R 4.0.5.

## 3. Results

### 3.1. Study Details and Demographics

**Figure 1:**
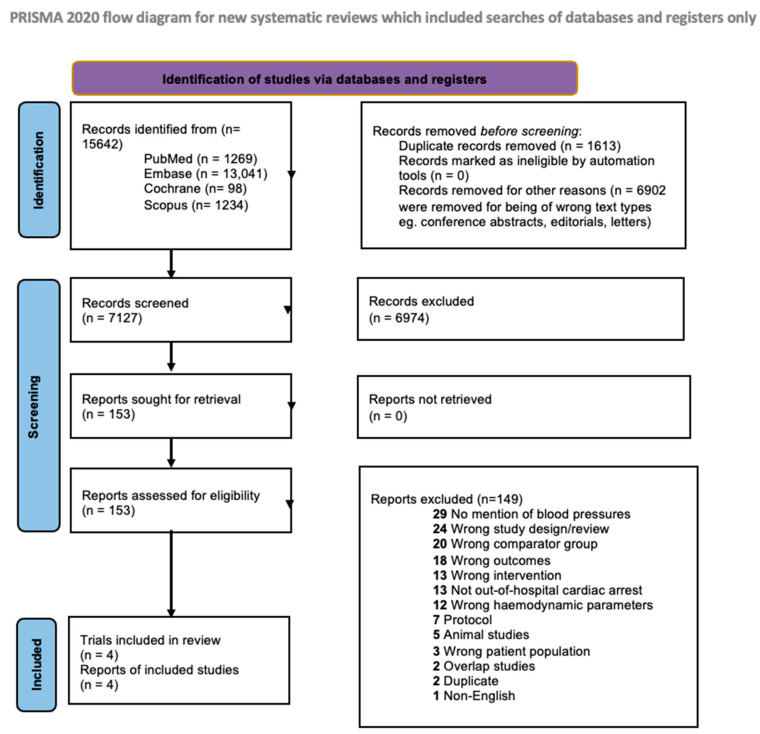
Flow diagram for Preferred Reporting Items for Systematic Reviews and Meta-Analyses.

Of 7127 references, we reviewed 153 full texts (Figure 1). Ultimately, four RCTs (533 patients with MAP ≤ 70 mmHg, 527 patients with MAP > 70 mmHg) met our inclusion criteria [13,14,15,29]. Of these four studies, two RCTs compared a target MAP of 65 mmHg with higher targets of 85–100 and 72 mmHg, respectively [13,29], and one compared a target MAP of 70 mmHg with higher targets of 80–100 mmHg [14], while the final study compared a target MAP of 63 mmHg to 77 mmHg [15]. The general characteristics of these studies can be found in Table 1. All studies involved centres from Europe; the mean age and the proportion of males between both groups were similar (Supplementary Table S4a–c). Other clinical characteristics are tabulated in Supplementary Table S5a,b. MAPs were recorded or maintained across a range of 36–48 h across RCTs, though there was also heterogeneity between each study’s protocol. Treatment details across studies are compiled in Supplementary Table S6a,b. 

### 3.2. Assessment of Study Quality

The risk of bias for the included studies is summarised in Supplementary Table S7a,b. In brief, all RCTs were all rated as either ‘low’ risk of bias or ‘some concerns’. The GRADE assessment of evidence is summarised in Supplementary Table S8. 

### 3.3. Primary Meta-Analysis

**Figure 2:**
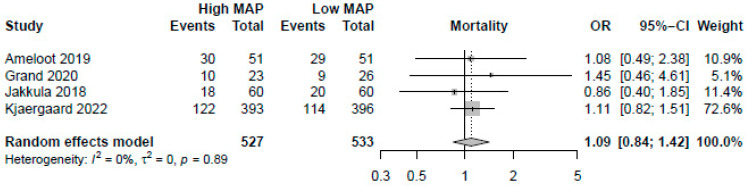
Forest plot for the odds ratios in mortality among resuscitated cardiac arrest patients [13,14,15,29]. For each study, the dot represents the overall effect estimate, the corresponding line represents the confidence intervals, and the grey box represents the weightage of each study.

**Figure 3:**
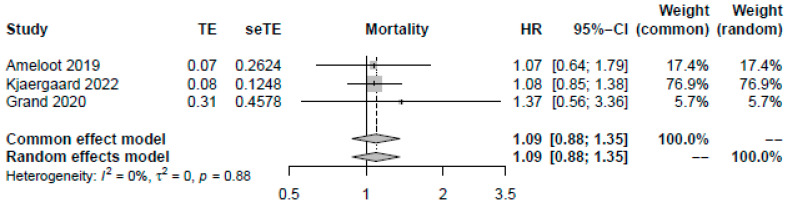
Forest plot for pooled available odds and hazard ratios reported by studies [13,15,29]. For each study, the dot represents the overall effect estimate, the corresponding line represents the confidence intervals, and the grey box represents the weightage of each study.

A higher MAP of >70 mmHg did not significantly improve mortality (OR: 1.09, 95%-CI: 0.84–1.42, *p* = 0.51, moderate certainty, Figure 2). Visual inspection of funnel plots did not reveal a likelihood of publication bias. Pooling the reported HRs from three RCTs yielded similar results (HR: 1.09, 95%-CI: 0.88–1.35, *p* = 0.42, Figure 3). Sensitivity analysis excluding the RCT with a median target of 70 mmHg also revealed no significant changes in survival (OR: 1.13, 95%-CI: 0.86–1.49, *p* = 0.40). As no studies were rated to have a high risk of bias, we did not conduct the sensitivity analysis excluding studies with a high risk of bias.

To account for the low number of RCTs, we included two observational studies [30,31] meeting our prespecified MAP criteria in another sensitivity analysis to improve the precision of our estimates (741 patients ≤ 70 mmHg; 1292 patients > 70 mmHg). Across eight pairwise comparisons in these six studies, a higher MAP did not improve mortality (OR: 0.85, 95%-CI: 0.60 to 1.22, *p* = 0.39). Other observational studies were excluded due to unsuitable MAP targets [32,33], inappropriate haemodynamic parameters [34], lack of mortality data [35,36], and overlaps of data [37]. We further explored the dose–response relationship of MAP on mortality using 70–80 mmHg and >80 mmHg as our thresholds. Using both 70–80 mmHg (2 studies, OR: 1.13, 95%-CI: 0.84–1.52, *p* = 0.41) and >80 mmHg (2 studies, OR: 0.96, 95%-CI: 0.55–1.68, *p* = 0.89) revealed no substantial differences to our results. 

Subgroup analysis did not find significant interaction effects based on the centre number (p_interaction_: 0.73), and duration of follow up (p_interaction_: 0.79 (Supplementary Table S9).

**Figure 4:**
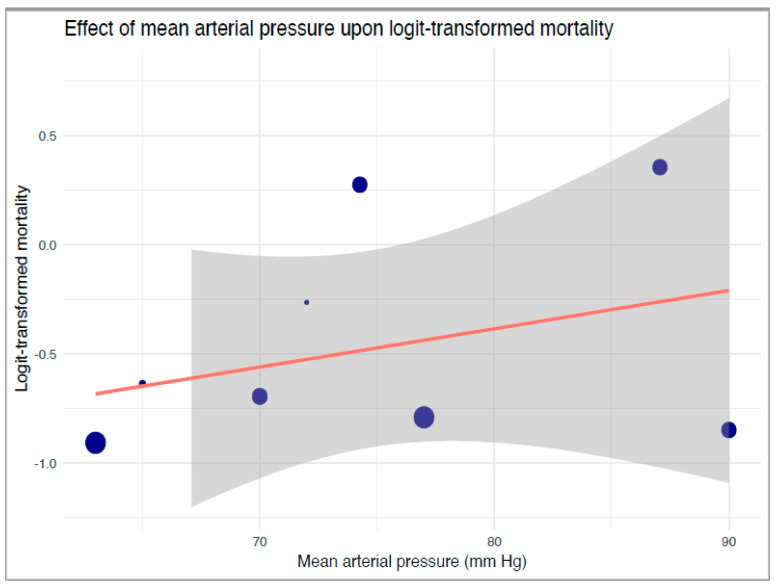
Robust variance estimate regression of the effect of mean arterial pressure upon logit-transformed mortality. The blue dots correspond to included studies and their relative sample sizes, the orange line denotes the regression, and grey boundaries denote the confidence intervals of the regression line.

Our robust variance strata-level meta-regression between strata-level logit-transformed proportions of mortality and reported mean MAP levels also revealed no significant associations between mean MAP and mortality (B: 0.017, 95%-CI: −0.073 to 0.11, *p* = 0.55, Figure 4). Our TSA of mortality revealed an extremely large RIS of 16,341 (Supplementary Table S10). Neither a statistically nor clinically significant reduction in mortality was noted in the primary analysis.

### 3.4. Secondary Outcomes

**Figure 5:**
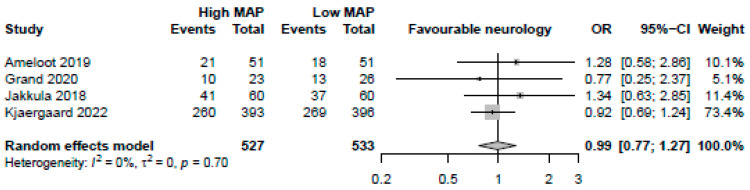
Forest plot for the odds ratio for favourable neurological outcome among resuscitated cardiac arrest patients [13,14,15,29]. For each study, the dot represents the overall effect estimate, the corresponding line represents the confidence intervals, and the grey box represents the weightage of each study.

MAP > 70 mmHg was neither significantly associated with favourable neurological assessments (4 studies, OR: 0.99, 95%-CI: 0.77–1.27 *p* = 0.92, moderate certainty, Figure 5) nor lower NSE levels (4 studies, MD: 0.55, 95%-CI: −1.67 to 2.78, *p* = 0.63, low certainty). MAP > 70 mmHg was not significantly associated with reductions in arrhythmia (2 studies, OR: 0.67, 95%-CI: 0.18–2.50, *p* = 0.56, low certainty) or AKIs (2 studies, OR: 0.74, 95%-CI: 0.27–2.03, *p* = 0.56, low certainty). There was a significant reduction in both days of mechanical ventilation (3 studies, MD: −0.91 days, 95%-CI: −1.51 to −0.31, *p* = 0.0029, high certainty) and ICU length of stay (3 studies, −0.78 days, 95%-CI: −1.54 to −0.021, *p* = 0.044, high certainty) with MAP > 70 mmHg. 

Trial sequential analysis for these outcomes (Supplementary Table S10) revealed that MAP > 70 mmHg only had a significant clinical benefit in reducing mechanical ventilation, reaching the required information size and crossing the TSA-adjusted boundary for benefit. Despite a statistically significant benefit, TSA demonstrated that the required information size was not reached in ICU length of stay. 

Forest plots for these outcomes are found in Supplementary Table S11, and a summary of all pooled outcomes can be found in Table 2. The details of other complications including bleeding, infection, and seizures are recorded in Supplementary Table S12a,b. 

In summary, our meta-analysis of four RCTs revealed that higher MAPs were not associated with reduced mortality or improved neurological outcomes, despite additional sensitivity analyses. Our robust variance strata-level meta-regression also revealed no significant associations between mean MAP and proportion of non-survivors, and trial sequential analysis revealed no meaningful survival benefit for higher MAPs. Aside from reducing ICU length of stay and mechanical ventilation time, were no significant benefits to higher MAPs in other secondary outcomes.

## 4. Discussion

In our meta-analysis of 1060 patients, we found that higher MAP targets of >70 mmHg were not associated with improved survival in resuscitated OHCA patients, nor did it affect neurological outcomes and incidence of arrhythmias and AKI. However, there were reductions in mechanical ventilation time and ICU stay. 

Neuroprotection after cardiac arrest involves balancing cerebral oxygen delivery and utilisation by optimising cerebral blood flow. In narrowed and right-shifted zones of autoregulation, higher MAP may ensure adequate cerebral perfusion [38]. Our findings stand in contrast to these theoretical benefits and prior reviews of observational studies [12], with neutral findings consistent across included RCTs [15] and trials on other forms of shock [39,40]. Selection and survivorship bias and baseline differences due to inadequate confounder adjustment may account for the discordance between observational and randomised data [33,41,42]. Indeed, our sensitivity analysis, which included observational studies, showed a trend towards improved mortality but was statistically not significant. Such findings serve to highlight the differences between observational studies and RCTs but also further confirm the nonsignificance between higher and lower MAPs. Our TSA of mortality not only revealed no significant benefit but also an extremely large RIS of over 16,000 needed to determine any meaningful benefit, which may not be feasible in this population. 

Whilst there is no consensus on what MAP targets improve outcomes, MAP targets between 72 mmHg–100 mmHg have been investigated [13,29]. Thus, our literature-based MAP threshold of 70mmHg was advantageous as it allowed us to comprehensively evaluate all available trials while ensuring consistency. Our sensitivity analysis exploring various MAP targets also suggested no benefit. However, several factors may have influenced this, including variability in MAP targets used in included RCTs leading to imprecision, duration of maintaining target MAPs (between 36 and 48 h), time to reach target MAP [14,15], and time from ROSC to randomisation, leading to inconsistency.

Resuscitated OHCA patients are heterogeneous, and cerebral dysregulation may manifest differently in each patient [38,43,44], therefore the influence of MAP on cerebral perfusion differs between patients and within each patient across different timepoints after ROSC. These patients may benefit from individualised, as opposed to static MAP targets, which may explain the neutral findings from our analysis of RCTs deploying static MAP targets. Future studies employing multimodal neuromonitoring after ROSC to guide individualised hemodynamic management are needed. 

One aspect that may be explored in further studies would be the impact of arrest aetiologies on subsequent haemodynamic management, something that has not been elicited in prior RCTs. It is possible that patients with cardiac arrest due to cardiac aetiologies such as acute coronary syndrome or heart failure may not benefit from higher MAP targets, as the increased vasopressor use may increase afterload and oxygen consumption of the heart, further aggravating myocardial injury [45]. Indeed, increased vasopressor use has been associated with higher mortality in myocardial infarction-induced cardiogenic shock [46]. On the other hand, arrests due to non-cardiac aetiologies may benefit from higher MAP targets, where the resuscitated heart might be able to cope with increased vasopressor use. Data stratified by aetiology of arrests were unfortunately not available in existing published trials, but this is an area of exploration that might inform the design of future trials. 

Our findings of reduced days of mechanical ventilation and ICU stay in higher MAPs, with overall benefit in days of mechanical ventilation affirmed by TSA, are worth discussing. It is possible that these findings are coincidental and related to the influence of mortality on such time-dependent variables, that is, individuals who died earlier did not have improved mortality but paradoxically had lower lengths of stay and ventilation times. However, the converse may also be true—a potential benefit of higher MAPs might be that they could improve organ perfusion and enhance recovery times in patients that do end up surviving the initial arrest. However, these findings may not be clinically meaningful given the lack of corroboration with improved mortality and neurological outcomes in other analyses.

As the first meta-analysis comparing MAP targets, this study provides a clearer picture of the existing evidence. Additionally, we also applied a robust search strategy validated by our medical information specialist, with comprehensive inclusion and exclusion criteria to reduce the risk of bias and confounders in analysis. By strictly including studies that met our criteria for MAP targets, we limited the concerns of heterogeneity between studies that affected prior systematic reviews and ensured consistency across our studies. We were also able to assess for potential sources of heterogeneity through subgroup analysis, and sensitivity analyses ensured results remained robust even when accounting for factors such as even higher MAP targets. 

Nevertheless, there are limitations to our study that require consideration. First, there are few well-conducted, well-powered RCTs, and our analysis may still lack power. While we attempted to address this by including observational studies to complement the randomised data, we could not include all observational studies due to our strict criteria, which was necessary to enhance consistency. Second, while we selected 70 mmHg as a threshold based on prior literature, there are no existing guidelines recommending a specific MAP target. This selection can be seen as at the discretion of the authors and can be interpreted as aggregating trials with non-homogenous MAP targets. We attempted to overcome these limitations by conducting sensitivity analyses assessing 70–80 and >80 mmHg, but outcomes for still higher MAP targets remain unknown. Finally, our results were all from centres in Europe, and may not represent patients from other regions where no data have been published. Given differences in healthcare practices and selection criteria, there may be differences in outcomes [47,48,49] 

In conclusion, the meta-analysis of higher MAP targets of above 70 mmHg shows that they are not significantly associated with improved survival and better neurological outcomes after cardiac arrest. While concordant with recent RCTs, significant limitations regarding existing research preclude strong or definitive conclusions from the results. Instead, these results provide a guide for further research, suggesting that evaluation of individualised (as opposed to static) blood pressure targets and other clinical parameters may be warranted. 

## Figures and Tables

**Table 1:** Summarised demographics table for included randomised controlled trials. [13,14,15,29] (Abbreviations: CPR: cardiopulmonary resuscitation, OHCA: out-of-hospital cardiac arrest, PEA: pulseless electrical activity, VF: ventricular fibrillation, VT: ventricular tachycardia).

Study	Continent	Hospitals	Location	Sample Size	Male Patients	Age (years), Mean ± SD	Arrest Location	Bystander CPR / Defibrillation	Presenting Rhythm
Ameloot 2019 [13]	Europe	2	OHCA	102 65 mmHg: 51 85-100 mmHg: 51	77 (75.5%) 38 39	65 ± 13 65 ± 12	Public: 26 Witnessed: 46 Public: 22 Witnessed: 44	26 30	VF: 30, VT: 2, PEA: 2, Asystole: 16 VF: 34, VT: 2 PEA: 4, Asystole: 11
Grand 2020 [29]	Europe	1	OHCA	49 65 mmHg: 26 72 mmHg: 23	43 (87.8%) 24 19	59 ± 13 63 ± 10	Witnessed 26 20	CPR: 24 Defibrillatiion: 4 CPR: 20 Defibrillation: 6	Shockable 23 22
Jakkula 2018 [14]	Europe	7	OHCA	120 65-75 mmHg: 60 80-100 mmHg: 60	98 (81.7%) 48 50	61 ± 11 58 ± 14	Home: 32 Public: 28 Home: 28 Public: 32	CPR 51 47	N/A
Kjaergaard 2022 [15]	Europe	2	OHCA	789 63 mmHg): 396 77 mmHg: 393	636 (80.6%) 320316	62 ± 14 63 ± 13	Witnessed 333 339	CPR: 339 Defibrillation: 84 CPR: 340 Defibrillation: 98	Shockable: 332 PEA: 14 Shockable: 335 PEA: 21

**Table 2:** Combined outcomes table for pooled estimates for mortality and pooled estimates for secondary outcomes. (Abbreviations: CI: confidence interval, HR: hazard ratio, mcg/L: microgram per litre, MD: mean difference, OR: odds ratio).

Outcome	Studies	Pairwise Comparisons	Pooled Estimate	95%-CI	*p*-Value	CERTAINTY OF EVIDENCE
**Primary outcome**						
Mortality	4	4	OR: 0.1.09	0.84 to 1.42	0.51	Moderate
Mortality including observational studies	6	8	OR: 0.85	0.60 to 1.22	0.39	-
Mortality for pooled HRs	3	3	HR: 1.09	0.88 to 1.35	0.42	-
**Secondary outcomes**						
Favourable neurological outcome	4	4	OR: 0.99	0.77 to 1.27	0.92	Moderate
Level of neuron-specific enolase (mcg/L)	4	4	MD: 0.55	−1.67 to + 2.78	0.63	Low
Arrhythmias	2	2	OR: 0.67	0.18 to 2.50	0.56	Low
Acute kidney injury	2	2	OR: 0.74	0.27 to 2.03	0.56	Low
Mechanical ventilation duration (days)	3	3	MD: −0.91	−1.51 to −0.31	0.0029	High
Intensive care unit length of stay (days)	3	3	MD: −0.78	−1.54 to −0.021	0.044	High

## Data Availability

All data generated or analysed during this study are included in the published studies and their supplementary information files.

## Supplementary Materials

The following supporting information can be downloaded at: https://www.mdpi.com/article/10.3390/jcm12134497/s1, Table S1. Preferred Reporting Items for Systematic Reviews and Meta-analyses checklist. Table S2. Search strategy for database. Table S3. Data collection template*. Table S4a. Pooled demographics of included trials. Table S4b. Demographics of included trials. Table S4c. Demographics of observational studies in sensitivity analysis. Table S5a. Details of cardiac arrest in included trials. Table S5b. Details of cardiac arrest of observational studies in sensitivity analysis. Table S6a. Details of treatment of included trials. Table S6b. Details of treatment of observational studies in sensitivity analysis. Table S7a. Risk of bias evaluation for included trials. Table S7b. Risk of bias evaluation for observational studies in sensitivity analysis. Table S8. Grading of Recommendations, Assessment, Development, and Evaluations. Table S9. Pooled data for subgroup analysis on mortality. Table S10. Trial sequential analysis for outcomes. Table S11. Forest plots for other secondary outcomes. Table S12a. Raw data for outcomes in included trials. Table S12b. Raw data for outcomes in observational studies in sensitivity analysis.
