# Supplementary material for: Blood Pressure Targets for Out-of-Hospital Cardiac Arrest: A Systematic Review and Meta-Analysis
Source: J Clin Med. 2023 Jul 5;12(13):4497. doi: 10.3390/jcm12134497 (PMC10342823; doi:10.3390/jcm12134497)
Supplement: Supplementary file 1 [file jcm-12-04497-s001.zip › jcm-2458084-supplementary.pdf]

Blood Pressure Targets for Out-of-Hospital Cardiac Arrest: A Systematic Review and Meta-Analysis

Shir Lynn Lim [1,2,3,*,†], Christopher Jer Wei Low [2,†], Ryan Ruiyang Ling [2], Rehena Sultana [4], Victoria Yang [5], Marcus E. H. Ong [6,7], Yew Woon Chia [2,8,9], Vijay Kumar Sharma [2,10] and Kollengode Ramanathan [2,11]

[1] Department of Cardiology, National University Heart Centre Singapore, Singapore 119074, Singapore
[2] Yong Loo Lin School of Medicine, National University of Singapore, Singapore 117597, Singapore;
e0474165@u.nus.edu (C.J.W.L.); ryanmedicine30@gmail.com (R.R.L.);
yew_woon_chia@ttsh.com.sg (Y.W.C.); vijay_kumar_sharma@nuhs.edu.sg (V.K.S.);
surrkr@nus.edu.sg (K.R.)
[3] Pre-Hospital Emergency Research Center, Duke-NUS Medical School, Singapore 169857, Singapore
[4] Centre for Quantitative Medicine, Duke-NUS Medical School, Singapore 169857, Singapore;
rehena.sultana@duke-nus.edu.sg
[5] Imperial College Healthcare NHS Trust, London W12 OHS, UK; victoria.yang@nhs.net
[6] Department of Emergency Medicine, Singapore General Hospital, Singapore 169608, Singapore; marcus.ong@duke-nus.edu.sg
[7] Health Services and Systems Research, Duke-NUS Medical School, Singapore 169857, Singapore
[8] Department of Cardiology, Tan Tock Seng Hospital, Singapore 308433, Singapore
[9] Lee Kong Chian School of Medicine, Nanyang Technological University, Singapore 639798, Singapore
[10] Division of Neurology, National University Health System, Singapore 119074, Singapore
[11] Cardiothoracic Intensive Care Unit, National University Heart Centre Singapore, Singapore 119074, Singapore
* Correspondence: mdclims@nus.edu.sg; Tel.: +65-6772-3301
† These authors contributed equally to this work.

Table of Contents

SUPPLEMENTARY TABLE S1: PREFERRED REPORTING ITEMS FOR SYSTEMATIC REVIEWS AND META-ANALYSES CHECKLIST 3

SUPPLEMENTARY TABLE S2: SEARCH STRATEGY FOR DATABASE 6

SUPPLEMENTARY TABLE S3: DATA COLLECTION TEMPLATE* 8

SUPPLEMENTARY TABLE S4a: POOLED DEMOGRAPHICS OF INCLUDED TRIALS 9

SUPPLEMENTARY TABLE S4b: DEMOGRAPHICS OF INCLUDED TRIALS 9

SUPPLEMENTARY TABLE S4c: DEMOGRAPHICS OF OBSERVATIONAL STUDIES IN SENSITIVITY ANALYSIS 10

SUPPLEMENTARY TABLE S5a: DETAILS OF CARDIAC ARREST IN INCLUDED TRIALS 12

SUPPLEMENTARY TABLE S5b: DETAILS OF CARDIAC ARREST OF OBSERVATIONAL STUDIES IN SENSITIVITY ANALYSIS 12

SUPPLEMENTARY TABLE S6a: DETAILS OF TREATMENT OF INCLUDED TRIALS 13

SUPPLEMENTARY TABLE S6b: DETAILS OF TREATMENT OF OBSERVATIONAL STUDIES IN SENSITIVITY ANALYSIS 14

SUPPLEMENTARY TABLE S7a: RISK OF BIAS EVALUATION FOR INCLUDED TRIALS 15

SUPPLEMENTARY TABLE S7b: RISK OF BIAS EVALUATION FOR OBSERVATIONAL STUDIES IN SENSITIVITY ANALYSIS 15

SUPPLEMENTARY TABLE S8: GRADING OF RECOMMENDATIONS, ASSESSMENT, DEVELOPMENT, AND EVALUATIONS 16

SUPPLEMENTARY TABLE S9: POOLED DATA FOR SUBGROUP ANALYSIS ON MORTALITY 18

SUPPLEMENTARY TABLE S10: TRIAL SEQUENTIAL ANALYSIS FOR OUTCOMES 19

SUPPLEMENTARY TABLE S11: FOREST PLOTS FOR OTHER SECONDARY OUTCOMES 23

SUPPLEMENTARY TABLE S12a: RAW DATA FOR OUTCOMES IN INCLUDED TRIALS 25

SUPPLEMENTARY TABLE S12b: RAW DATA FOR OUTCOMES IN OBSERVATIONAL STUDIES IN SENSITIVITY ANALYSIS 26

Supplementary Table S1: Preferred Reporting Items for Systematic Reviews and Meta-analyses checklist

Section and Topic	Item #	Checklist item	Location where item is reported
**TITLE**			
Title	1	Identify the report as a systematic review.	Page 1
**ABSTRACT**			
Abstract	2	See the PRISMA 2020 for Abstracts checklist.	Page 3
**INTRODUCTION**			
Rationale	3	Describe the rationale for the review in the context of existing knowledge.	Page 5
Objectives	4	Provide an explicit statement of the objective(s) or question(s) the review addresses.	Page 5
**METHODS**			
Eligibility criteria	5	Specify the inclusion and exclusion criteria for the review and how studies were grouped for the syntheses.	Page 6
Information sources	6	Specify all databases, registers, websites, organisations, reference lists and other sources searched or consulted to identify studies. Specify the date when each source was last searched or consulted.	Page 6
Search strategy	7	Present the full search strategies for all databases, registers and websites, including any filters and limits used.	Supplementary Table 3
Selection process	8	Specify the methods used to decide whether a study met the inclusion criteria of the review, including how many reviewers screened each record and each report retrieved, whether they worked independently, and if applicable, details of automation tools used in the process.	Page 6-7
Data collection process	9	Specify the methods used to collect data from reports, including how many reviewers collected data from each report, whether they worked independently, any processes for obtaining or confirming data from study investigators, and if applicable, details of automation tools used in the process.	Page 6-7
Data items	10a	List and define all outcomes for which data were sought. Specify whether all results that were compatible with each outcome domain in each study were sought (e.g. for all measures, time points, analyses), and if not, the methods used to decide which results to collect.	Page 6-8
	10b	List and define all other variables for which data were sought (e.g. participant and intervention characteristics, funding sources). Describe any assumptions made about any missing or unclear information.	Page 6-8 Supplementary Table 2
Study risk of bias assessment	11	Specify the methods used to assess risk of bias in the included studies, including details of the tool(s) used, how many reviewers assessed each study and whether they worked independently, and if applicable, details of automation tools used in the process.	Page 7
Effect measures	12	Specify for each outcome the effect measure(s) (e.g. risk ratio, mean difference) used in the synthesis or presentation of results.	Page 7
Synthesis methods	13a	Describe the processes used to decide which studies were eligible for each synthesis (e.g. tabulating the study intervention characteristics and comparing against the planned groups for each synthesis (item #5)).	Page 6-8

	13b	Describe any methods required to prepare the data for presentation or synthesis, such as handling of missing summary statistics, or data conversions.	Page 7-8
	13c	Describe any methods used to tabulate or visually display results of individual studies and syntheses.	Page 7-8, Supplementary Tables 4-6
	13d	Describe any methods used to synthesize results and provide a rationale for the choice(s). If meta-analysis was performed, describe the model(s), method(s) to identify the presence and extent of statistical heterogeneity, and software package(s) used.	Page 7-8
	13e	Describe any methods used to explore possible causes of heterogeneity among study results (e.g. subgroup analysis, meta-regression).	Page 7
	13f	Describe any sensitivity analyses conducted to assess robustness of the synthesized results.	Page 7
Reporting bias assessment	14	Describe any methods used to assess risk of bias due to missing results in a synthesis (arising from reporting biases).	Page 7, Supplementary Table 7a and 7b
Certainty assessment	15	Describe any methods used to assess certainty (or confidence) in the body of evidence for an outcome.	Page 7, Supplementary Table 8
**RESULTS**			
Study selection	16a	Describe the results of the search and selection process, from the number of records identified in the search to the number of studies included in the review, ideally using a flow diagram.	Page 8
	16b	Cite studies that might appear to meet the inclusion criteria, but which were excluded, and explain why they were excluded.	Page 8-9
Study characteristics	17	Cite each included study and present its characteristics.	Page 8, Supplementary Tables 4-6
Risk of bias in studies	18	Present assessments of risk of bias for each included study.	Page 9, Supplementary Table 7a and 7b
Results of individual studies	19	For all outcomes, present, for each study: (a) summary statistics for each group (where appropriate) and (b) an effect estimate and its precision (e.g. confidence/credible interval), ideally using structured tables or plots.	Page 9-10, Table 1
Results of syntheses	20a	For each synthesis, briefly summarise the characteristics and risk of bias among contributing studies.	Page 8-10, Supplementary Table 4 to 7b
	20b	Present results of all statistical syntheses conducted. If meta-analysis was done, present for each the summary estimate and its precision (e.g. confidence/credible interval) and measures of statistical heterogeneity. If comparing groups, describe the direction of the effect.	Page 9-10, Table 1
	20c	Present results of all investigations of possible causes of heterogeneity among study results.	Page 9-10
	20d	Present results of all sensitivity analyses conducted to assess the robustness of the synthesized results.	Page 9-10

Reporting biases	21	Present assessments of risk of bias due to missing results (arising from reporting biases) for each synthesis assessed.	Supplementary Table 7a and 7b
Certainty of evidence	22	Present assessments of certainty (or confidence) in the body of evidence for each outcome assessed.	Supplementary Table 8
**DISCUSSION**			
Discussion	23a	Provide a general interpretation of the results in the context of other evidence.	Page 11-13
	23b	Discuss any limitations of the evidence included in the review.	Page 12-13
	23c	Discuss any limitations of the review processes used.	Page 12-13
	23d	Discuss implications of the results for practice, policy, and future research.	Page 12-13
**OTHER INFORMATION**			
Registration and protocol	24a	Provide registration information for the review, including register name and registration number, or state that the review was not registered.	Page 6
	24b	Indicate where the review protocol can be accessed, or state that a protocol was not prepared.	Page 6
	24c	Describe and explain any amendments to information provided at registration or in the protocol.	Page 6
Support	25	Describe sources of financial or non-financial support for the review, and the role of the funders or sponsors in the review.	Page 14
Competing interests	26	Declare any competing interests of review authors.	Page 14-15
Availability of data, code and other materials	27	Report which of the following are publicly available and where they can be found: template data collection forms; data extracted from included studies; data used for all analyses; analytic code; any other materials used in the review.	Page 14-15

From: Page MJ, McKenzie JE, Bossuyt PM, Boutron I, Hoffmann TC, Mulrow CD, et al. The PRISMA 2020 statement: an updated guideline for reporting systematic reviews. BMJ 2021;372:n71. doi: 10.1136/bmj.n71

Supplementary Table S2: Search strategy for database

Pubmed

Search no.	Query	Result
1	Out-of-hospital cardiac arrest[MeSH] OR Cardiopulmonary Resuscitation[MeSH] OR ((out-of-hospital[Title/Abstract]) AND (cardiac arrest[Title/Abstract])) OR cardiopulmonary arrest[Title/Abstract] OR resuscitation from cardiac arrest[Title/Abstract] OR cardiopulmonary resuscitation[Title/Abstract]	35,169
2	Blood pressure[MeSH] OR Arterial pressure[MeSH] OR systolic blood pressure*[Title/Abstract] OR arterial pressure*[Title/Abstract]	385,724
3	#1 AND #2	1,269

Embase

Search no.	Query	Results
1	'out of hospital cardiac arrest'/exp OR 'cardiopulmonary resuscitation'/exp OR ('out-of-hospital' NEAR/3 'cardiac arrest'):ti,ab OR 'cardiopulmonary arrest':ti,ab OR 'resuscitation from cardiac arrest':ti,ab OR 'cardiopulmonary resuscitation':ti,ab	139,479
2	'blood pressure'/exp OR 'arterial pressure'/exp OR 'systolic blood pressure':ti,ab OR 'arterial pressure':ti,ab	707,343
3	#1 AND #2	13,041

Cochrane

Search no.	Query	results
1	MeSH descriptor: [Out-of-Hospital Cardiac Arrest] explode all trees	521
2	MeSH descriptor: [Cardiopulmonary Resuscitation] explode all trees	1,247
3	((out-of-hospital)ti,ab NEAR/3 (cardiac arrest)ti,ab) OR (cardiopulmonary arrest OR resuscitation from cardiac arrest OR cardiopulmonary resuscitation)ti,ab	2,888
4	MeSH descriptor: [Blood Pressure] explode all trees	28,794
5	MeSH descriptor: [Arterial Pressure] explode all trees	481
6	(systolic blood pressure OR arterial pressure)ti,ab	760
7	(#1 OR #2 OR #3) AND (#4 OR #5 OR #6)	98

Scopus

Search no.	Query	results
1.	TITLE-ABS((“out-of-hospital” AND “cardiac arrest”) OR “cardiopulmonary arrest” OR “resuscitation from cardiac arrest” OR “cardiopulmonary resuscitation”)	28,441
2.	TITLE-ABS (“blood pressure” OR “arterial pressure”)	452,481
3.	#1 AND #2	1,234

Supplementary Table S3: Data collection template*

Study characteristics: Study authors, date of publication, study centres/countries, duration of study, inclusion/exclusion criteria, MAP levels assessed

Patient demographics: Sample size, age, number of males, body mass index, cardiac history (prior percutaneous coronary intervention, prior coronary artery bypass, prior myocardial infarction and other relevant cardiac comorbidities), other comorbidities (hypertension, diabetes mellitus, hyperlipidaemia, obesity, chronic obstructive pulmonary disease, smoking, any other comorbidities reported by studies)

Cardiac arrest characteristics: Aetiology of arrest, bystander resuscitation, presenting rhythm, time to return of spontaneous circulation, no flow time, low flow time, mean arterial pressure on admission, Sequential Organ Failure Assessment score, other cardiac arrest characteristics as available

Treatment characteristics: Angiography done, percutaneous coronary intervention done, Targeted Temperature Management characteristics, inotropes used, complete revascularisation achieved

Primary outcomes: Mortality

Secondary outcomes: Favourable neurological outcome, arrhythmia, neuron specific enolase levels, other post-cardiac arrest complications as available

*: Not all prespecified data was available from the studies, and data was extracted where available

Supplementary Table S4a: Pooled demographics of included trials

	Higher MAP	Lower MAP
**Pooled mean age/years**	62.37 (95%-CI: 59.89 to 64.85)	61.97 (95%-CI: 60.27 to 63.68)
**Proportion of males/%**	80.46 (95%-CI: 76.85 to 83.62)	80.68 (95%-CI: 77.10 to 83.81)

Supplementary Table S4b: Demographics of included trials

Study	Continent	Hospitals	Location	Sample Size	Male Patients	Age (years), Mean ± SD	Weight (kg), Mean ± SD	Cardiac History	Other Comorbidities
**Ameloot 2019**	Europe	2	OHCA	102	77 (75.5%)		N/A		
				Low (65 mmHg): 51	38	65 ± 13		MI: 4, CS: 1, HF: 5	HTN: 22, DM: 3, COPD: 5, CKD: 5, Stroke: 3
				High (85-100 mmHg): 51	39	65 ± 12		MI: 7, CS: 5, HF: 7	HTN: 21, DM: 7, COPD: 8, CKD: 7, Stroke: 3
**Grand 2020**	Europe	1	OHCA	49	43 (87.8%)		N/A		
				Low (65 mmHg): 26	24	59 ± 13		CCF: 3, CAD: 6 PCI: 2, CABG: 0,	HTN: 10, Stroke: 2, Pulmonary disease: 0, Nephropathy: 6
				High (72 mmHg): 23	19	63 ± 10		CCF: 1, CAD: 5, PCI: 1, CABG: 2,	HTN: 10, Stroke: 3, Pulmonary disease: 2, Nephropathy: 5
**Jakkula 2018**	Europe	7	OHCA	120	98 (81.7%)				
				Low (65-75 mmHg): 60	48	61 ± 11	86 ± 19	NYHA 4 HF: 0	HTN: 26, Asthma: 5, Smoker: 20
				High (80-100 mmHg): 60	50	58 ± 14	83 ± 14	NYHA 4 HF: 2	HTN: 34, Asthma: 3, Smoker: 20
**Kjaergaard 2022**	Europe	2	OHCA	789	636 (80.6%)		N/A		
				Low (63 mmHg): 396	320	62 ± 14		MI: 78, AF: 60, HF: 72	HTN: 186, DM: 62, COPD: 33, Stroke: 36, CKD: 17, RRT: 2
				High (77 mmHg): 393	316	63 ± 13		MI: 94, AF: 67, HF: 65	HTN: 176, DM: 48, COPD: 30, Stroke: 23, CKD: 22, RRT: 2

Supplementary Table S4c: Demographics of observational studies in sensitivity analysis

Study	Continent	Hospitals	Location	Sample Size	Male Patients	Age (years), Mean ± SD	Weight (kg), Mean ± SD	Cardiac History	Other Comorbidities
**Grand 2019**	Europe Australia	36	OHCA	851	699 (82.1%)		N/A		
				Low (<70 mmHg): 188	162	66 ± 12		CAD: 54, MI: 45, CA: 3, CCF: 13	HTN: 61, DM: 29, Asthma/COPD: 15, RRT: 2, Stroke/TIA: 16, Alcoholism: 1
				High (70-80 mmHg): 364	299	63 ± 13		CAD: 105, MI: 88, CA: 9, CCF: 26	HTN: 139, DM: 49, Asthma/COPD: 40, RRT: 3, Stroke/TIA: 25, Alcoholism: 16
				High (>80 mmHg): 299	238	63 ± 12		CAD: 68, MI: 46, CCF: 17	HTN: 134, DM: 44, Asthma/COPD: 31, RRT: 1, Stroke/TIA: 25, Alcoholism: 13
**Russo 2017**	North America	1	OHCA	122	93 (76.2%)				
				Low (<70 mmHg): 20	10	66 ± 12	81 ± 16	MI: 4, PCI: 4, CABG: 0	HTN: 13, DM: 3, DLD: 10, Stroke/TIA: 2, Smoker: 7
				High (70 to <80 mmHg): 67	55	58 ± 14	87 ± 18	MI: 13, PCI: 4, CABG: 1	HTN: 28, DM: 14, DLD: 27, Stroke/TIA: 3, Smoker: 28
				High (≥ 80 mmHg): 35	28	56 ± 11	85 ± 18	MI: 6, PCI: 3, CABG: 2	HTN: 16, DM: 4, DLD: 13, Stroke/TIA: 2, Smoker: 17

Note: CI, confidence intervals; MAP, mean arterial pressure; *Low and High, treatment groups with low and high MAP targets; MI, myocardial infarction; CS, cardiac surgery; HF, heart failure; HTN, hypertension; DM, diabetes mellitus; COPD, chronic obstructive pulmonary disease; CKD, chronic kidney disease; CAD, coronary artery disease; CA, cardiac arrest; CCF, congestive cardiac failure; RRT, renal replacement therapy; TIA, transient ischaemic attack; NYHA 4 HF, NYHA Class IV heart failure; AF, atrial fibrillation; PCI, percutaneous coronary intervention; CABG, coronary artery bypass graft; DLD, dyslipidaemia.

Supplementary Table S5a: Details of cardiac arrest in included trials

Study	MAP group/mmHg	Arrest Location	Bystander CPR / Defibrillation	Time to CPR (min), Median (IQR)	Presenting Rhythm	Time to ROSC (min), Median (IQR) or Mean ± SD	Arrest Aetiology	MAP on admission (mmHg), Mean ± SD
**Ameloot 2019**				N/A				
	Low (65 mmHg)	Public: 26<br>Witnessed: 46	26		VF: 30<br>VT: 2<br>PEA: 2<br>Asystole: 16	17 (11.5-25)	STEMI: 23 NSTEMI: 8<br>Arrhythmia: 11<br>Hypoxia: 4<br>Other/Unclear: 4	84 ± 26
	High (85-100 mmHg)	Public: 22<br>Witnessed: 44	30		VF: 34<br>VT: 2<br>PEA: 4<br>Asystole: 11	18 (12-25)	STEMI: 22 NSTEMI: 6<br>Arrhythmia: 14<br>Hypoxia: 7<br>Other/Unclear: 2	88 ± 21
**Grand 2020**		<u>Witnessed</u>		N/A	<u>Shockable</u>		<u>ST elevation or BBB on admission</u>	N/A
	Low (65 mmHg)	26	CPR: 24<br>Defibrillatiion: 4		23	16 (12-36)	18	
	High (72 mmHg)	20	CPR: 20<br>Defibrillation: 6		22	18 (13-21)	19	
**Jakkula 2018**	120		<u>CPR</u>		N/A		N/A	N/A
	Low (65-75 mmHg)	Home: 32<br>Public: 28	51	To BLS: 8 (6-10)<br>To ALS:10 (7-12)		22 (16-27)		
	High (80-100 mmHg)	Home: 28<br>Public: 32	47	To BLS: 7 (5-9)<br>To ALS: 10 (7-12)		19 (15-25)		
**Kjaergaard 2022**		<u>Witnessed</u>		N/A			<u>STEMI</u>	N/A
	Low (63 mmHg)	333	CPR: 339<br>Defibrillation: 84		Shockable: 332<br>PEA: 14	21 ± 15	178	
	High (77 mmHg)	339	CPR: 340<br>Defibrillation: 98		Shockable: 335<br>PEA: 21	21 ± 13	172	

Supplementary Table S5b: Details of cardiac arrest of observational studies in sensitivity analysis

Study	MAP group/mmHg	Arrest Location	Bystander CPR / Defibrillation	Time to CPR (min), Median (IQR)	Presenting Rhythm	Time to ROSC (min), Median (IQR) or Mean ± SD	Arrest Aetiology	MAP on admission (mmHg), Mean ± SD
**Grand 2019**		Witnessed		N/A	Shockable		ST elevation on admission	N/A
	Low (<70 mmHg)	174	CPR: 145<br>Defib: 18		161	25 (17-38)	108	
	High (70-80 mmHg)	331	CPR: 271<br>Defib: 36		298	25 (16-37)	142	
	High (>80 mmHg)	147	CPR: 208 Defib: 32		227	25 (16-39)	105	
**Russo 2017**	122	Witnessed	CPR	N/A	N/A		STEMI	N/A
	Low (<70 mmHg)	17	13			24 (13-30)	10	
	High (70 to <80 mmHg)	54	39			21 (14-30)	36	
	High (≥ 80 mmHg): 35	28	19			19 (14-29)	16	

Note. *Low, Medium and High, treatment groups with low, medium and high MAP targets; MAP, mean arterial pressure; CPR, cardiopulmonary resuscitation; ROSC, return of spontaneous circulation; SD, standard deviation; VF, ventricular fibrillation; VT, ventricular tachycardia; PEA, pulseless electrical activity; STEMI, ST-elevation myocardial infarction; NSTEMI, non-ST-elevation myocardial infarction; BBB, bundle branch block; BLS, basic life support; ALS, advanced life support.

Supplementary Table S6a: Details of treatment of included trials

Study	MAP group/ mmHg*	Duration MAP targets were recorded or maintained	Angiography done	PCI done	Inotropes given	TTM	Others
Ameloot 2019	Low: 65	Maintained for 36 hour intervention period upon ICU admission	44/51	28/51	Fluids, inotropes and vasopressors were at the discretion of treating physicians	Therapeutic hypothermia was administered in all patients by endovascular or or surface cooling systems, at 33 celsius for 24 hours, with rewarming at 0.3 degrees celsius per hour until 36 degrees celsius	
	High: 85 to 100		45/51	27/51	Fluids, inotropes and vasopressors were given with a predefined flow-chart to target an MAP of 85-100 mmHg		
Grand 2020	Low: 65	Maintained for a 48 hour intervention period from randomisation	25/26	11/26	Dose of noradrenaline: 1013 (488-4803) pg/ml	Target temperature was induced and maintained at 36 degrees Celsius for 24 hour after randomisation, followed by active rewarning of no more than 0.5 degrees celsius per hour to 37 degrees celsius	Dose of prehospital adrenaline: 1.5 (0-3) mg
	High: 72		21/23	15/23	Dose of noradrenaline: 2939 (699-4837) pg/ml		Dose of prehospital adrenaline: 1 (0-2) mg
Jakkula 2018	Low: 65 to 75	Maintained for 36 hour intervention period upon ICU admission	35/60		Dose of noradrenaline: 0.06 ± 0.08 ug/kg/min	33 degrees celsius: 42<br>36 degrees celsius: 18	Prehospital thrombolysis: 3<br>Prehospital cooling: 4<br>Intubated during resuscitation: 26
	High: 80 to 100		28/60		Dose of noradrenaline: 0.08 ± 0.11 ug/mg/min	33 degrees celsius: 41<br>36 degrees celsius: 19	Prehospital thrombolysis: 1<br>Prehospital cooling: 6<br>Intubated during resuscitation: 31
Kjaergaard	Low: 63	Maintained for 48 hour intervention period from randomisation	358/396	165/396	Norepinephrine infusions and dopamine infusion for a maximal dose of 10ug/kg/min	Targeted temperature management at 36 degrees celsius for 24 hours, with subsequent rewarming of less than 0.5 degrees celsius per hour	
	High: 77		364/393	171/393			

Supplementary Table S6b: Details of treatment of observational studies in sensitivity analysis

Study	MAP group/ mmHg*	Duration MAP targets were recorded or maintained	Angiography done	PCI done	Inotropes given	TTM	Others
Grand 2019	Low: <70	Recorded up till 28 hours after randomisation at 0, 4, 12, 20 and 28 hours	138/188	100/188	High vasopressor (dopamine and norepinephrine) need: 91	Target temperature was induced and maintained for 28 hour after randomisation, followed by active rewarning of no more than 0.5 degrees celsius per hour to 37 degrees celsius	
	High: 70 to 80 (75)		223/364	160/364	High vasopressors need: 172		
	High: >80		179/299	117/299	High vasopressors need: 125		
Russo 2017	Low: <70	Recorded for 96 hours following ICU admission	17/20		Prolonged vasoactive agent use: 15 Prolonged high dose vasoactive agent use: 14	Targeted temperature management for 96 hours	Transfer to cardiac ICU from neighbouring hospital: 12
	High: 70 to <80		55/67		Prolonged vasoactive agent use: 51 Prolonged high dose vasoactive agent use: 30		Transfer to cardiac ICU from neighbouring hospital: 47
	High: ≥ 80		29/35		Prolonged vasoactive agent use: 12 Prolonged high dose vasoactive agent use: 29		Transfer to cardiac ICU from neighbouring hospital: 25

Note: MAP, mean arterial pressure; PCI, percutaneous coronary intervention; TTM, targeted temperature management; ICU, intensive care unit;

Supplementary Table S7a: Risk of bias evaluation for included trials

Author	Risk of bias arising from the randomisation process	Deviations from the intended interventions	Missing outcome data	Risk of bias in measurement of the outcome	Risk of bias in selection of the reported result	Other sources	Overall
Ameloot 2019	Low risk	Low risk	Low risk	Low risk	Low risk	Low risk	Low risk
Grand 2020	Low risk	Low risk	Low risk	Low risk	Low risk	Low risk	Low risk
Jakkula 2018	Low risk	Low risk	Low risk	Some concerns	Low risk	Low risk	Some concerns
Kjaegaard 2022	Low risk	Low risk	Low risk	Low risk	Low risk	Low risk	Low risk

Supplementary Table S7b: Risk of bias evaluation for observational studies in sensitivity analysis

	SELECTION DOMAIN				COMPARABILITY DOMAIN	OUTCOME DOMAIN				
**Author**	**Representative-ness of exposed cohort**	**Selection of non-exposed cohort**	**Ascertainment of exposure**	**Demonstration that outcome of interest was not present at start of the study**	**Comparability of cohorts on the basis of the design or analysis controlled for confounders**	**Assessment of outcome**	**Was follow up long enough for outcomes to occur (state the median duration of follow up and a brief rationale as well)**	**Adequacy of follow up of cohorts**	**Newcastle Ottawa Scale Score**	**Overall**
Grand 2019	X	X	X	X		X	X	X	7	High risk
Russo 2017	X	X	X	X		X	X	X	7	High risk

Supplementary Table S8: Grading of Recommendations, Assessment, Development, and Evaluations

Certainty assessment							№ of patients		Effect		Certainty	Importance
№ of studies	Study design	Risk of bias	Inconsistency	Indirectness	Imprecision	Other considerations	higher MAP targets	lower MAP targets	Relative (95% CI)	Absolute (95% CI)		
**In-hospital mortality (follow-up: 180 days; assessed with: OR)**												
4	randomised trials	not serious	not serious	not serious	serious[a]	none	180/527 (34.2%)	172/533 (32.3%)	**OR 1.09** (0.84 to 1.42)	**19 more per 1,000** (from 37 fewer to 81 more)	⊕⊕⊕○ Moderate	CRITICAL
**Favourable neurological outcome (follow-up: 180 days; assessed with: OR)**												
4	randomised trials	not serious	not serious	not serious	serious[b]	none	332/527 (63.0%)	337/533 (63.2%)	**OR 0.99** (0.77 to 1.27)	**2 fewer per 1,000** (from 63 fewer to 54 more)	⊕⊕⊕○ Moderate	CRITICAL
**Neuron specific enolase levels (assessed with: mean difference)**												
4	randomised trials	not serious	not serious	not serious	very serious[c]	none	527	533	-	MD **0.32 mcg/L higher** (1.9 lower to 2.53 higher)	⊕⊕○○ Low	IMPORTAN T
**Arrhythmia (assessed with: OR)**												
2	randomised trials	not serious	serious[d]	not serious	serious[e]	none	66/444 (14.9%)	67/447 (15.0%)	**OR 0.67** (0.18 to 2.50)	**44 fewer per 1,000** (from 119 fewer to 156 more)	⊕⊕○○ Low	IMPORTAN T
**Acute kidney injury (assessed with: OR)**												
2	randomised trials	not serious	serious[f]	not serious	Serious[g]	none	44/416 (10.6%)	48/422 (11.4%)	**OR 0.74** (0.27 to 2.03)	**27 fewer per 1,000** (from 80 fewer to 93 more)	⊕⊕○○ Low	IMPORTAN T
**Days of mechanical ventilation (assessed with: mean difference)**												
3	randomised trials	not serious	not serious	not serious	not serious	none	134	137	-	MD **0.91 days fewer** (1.51 fewer to 0.31 fewer)	⊕⊕⊕⊕ High	IMPORTAN T

Certainty assessment							№ of patients		Effect		Certainty	Importance
№ of studies	Study design	Risk of bias	Inconsistency	Indirectness	Imprecision	Other considerations	higher MAP targets	lower MAP targets	Relative (95% CI)	Absolute (95% CI)		
**Days of intensive care unit stay (assessed with: mean difference)**												
3	randomised trials	not serious	not serious	not serious	not serious	none	134	137	-	MD **0.78 days fewer** (1.54 fewer to 0.02 fewer)	⊕⊕⊕⊕ High	IMPORTAN T

CI: confidence interval; **MD:** mean difference; **OR:** odds ratio

Explanations

a. Confidence intervals (0.84 to 1.42) crossed the null effect of 1.
b. Confidence intervals (0.77 to 1.27) crossed the null effect of 1.
c. Confidence intervals of included studies demonstrated very serious imprecision, with values ranging from -30 mcg/L to +19 mcg/L, and also crossed null effect of 1.
d. Heterogeneity was 84%, and there was significant variability between studies on the forest plots.
e. Confidence intervals (0.18 to 2.50) crossed the null effect of 1 and differed from pooled estimates greatly.
f. Heterogeneity was 51%, and there was significant variability between studies on the forest plots.
g. Confidence intervals (0.27 to 2.03) crossed the null effect of 1 and differed from pooled estimates greatly

Supplementary Table S9: Pooled data for subgroup analysis on mortality

Subgroup		Pairwise comparisons	Odds ratio	95%-CI
Centre number<br>$P_{interaction}$ = 0.73	Single centre	1	1.45	0.46 to 4.61
	Two centre	2	1.11	0.84 to 1.47
	Multicentre	1	0.86	0.40 to 1.85
Duration of follow up<br>$P_{interaction}$ = 0.79	30 days	1	0.86	0.40 to 1.85
	90 days	1	1.11	0.82 to 1.51
	180 days	2	1.19	0.62 to 2.28

Note: CI, confidence interval

Supplementary Table S10: Trial sequential analysis for outcomes

Graph of outcome	Interpretation
Mortality<br>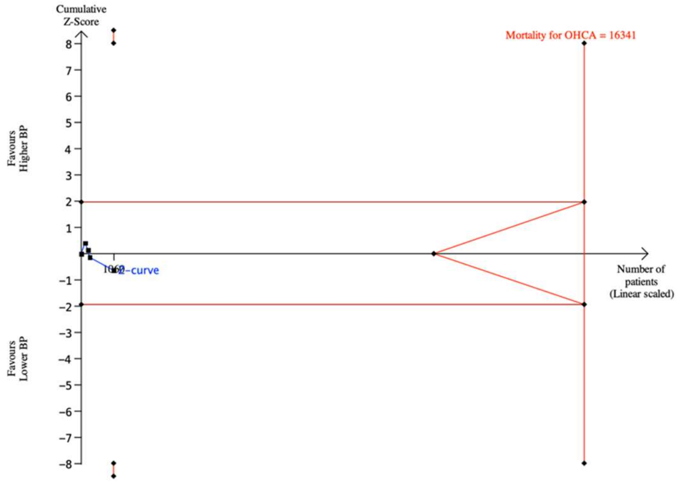	Trial sequential analysis for mortality between both groups of patients. The required information size is 16341, which is not achieved.<br><br>The cumulative Z-curve does not cross the conventional (straight dark red line) and TSA-adjusted boundaries (bright red line) for benefit, showing no statistical or clinical benefit in reducing mortality.
Favourable neurological outcome<br>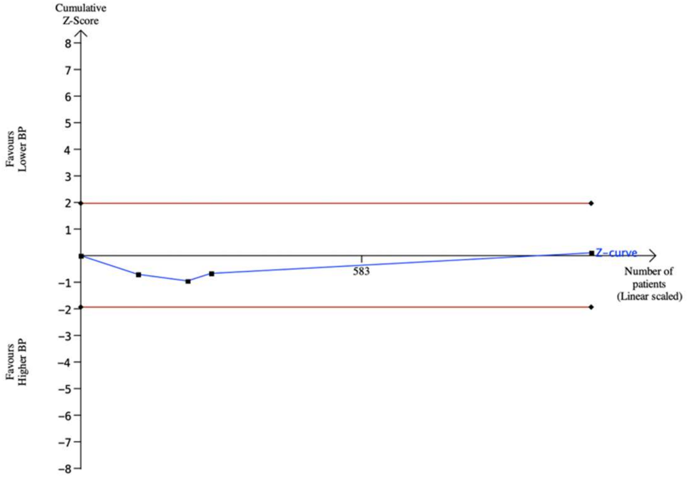	Trial sequential analysis for mortality between both groups of patients. Due to insufficient available data, the required information size is too high such that it cannot be estimated based on the graph.<br><br>The cumulative Z-curve does not cross the conventional (straight dark red line) and TSA-adjusted boundaries (bright red line) for benefit, showing no statistical or clinical benefit in reducing favourable neurological outcome.

Neuron-specific enolase levels<br>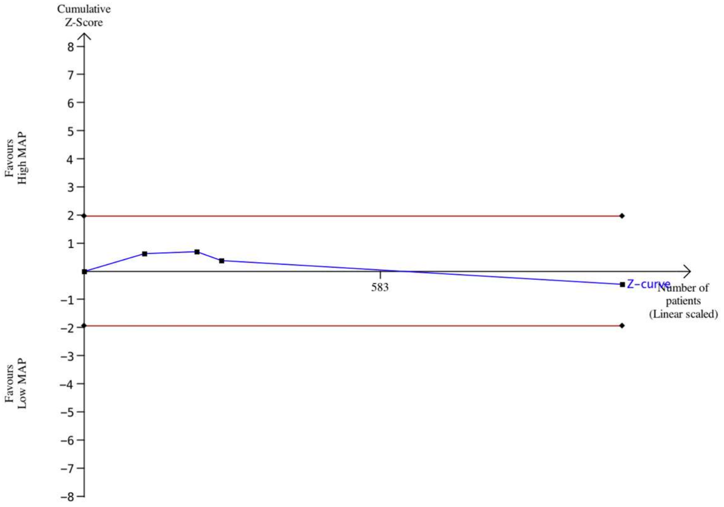 	Trial sequential analysis for mortality between both groups of patients. Due to insufficient available data, the required information size is too high such that it cannot be estimated based on the graph.<br><br>The cumulative Z-curve does not cross the conventional (straight dark red line) and TSA-adjusted boundaries (bright red line) for benefit, showing no statistical or clinical benefit in reducing levels of neuron-specific enolase.
Arrhythmia	Trial sequential analysis for mortality between both groups of patients. The required information size is 6268, which is not achieved.<br><br>The cumulative Z-curve does not cross the conventional (straight dark red line) and TSA-adjusted boundaries (bright red line) for benefit, showing no statistical or clinical benefit in reducing acute kidney injury

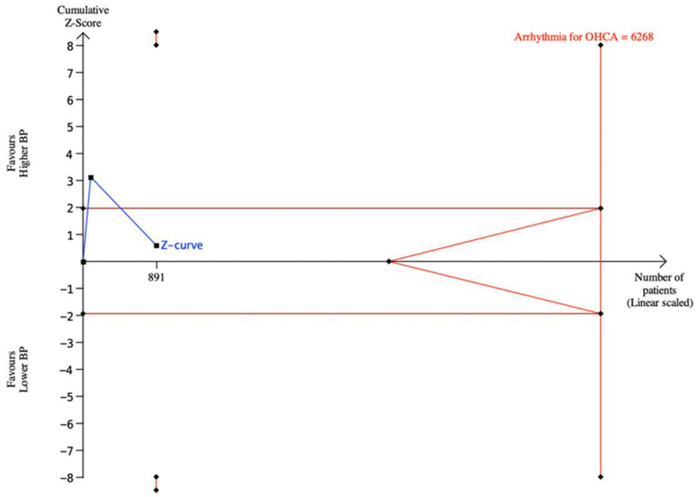 	
Acute kidney injury<br>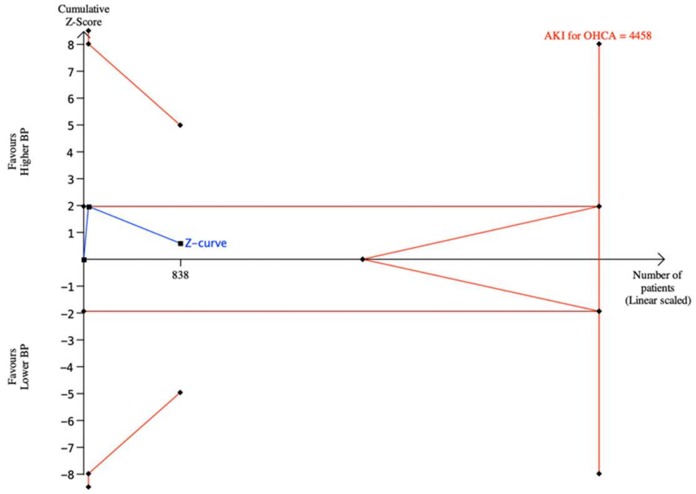 	Trial sequential analysis for mortality between both groups of patients. The required information size is 4458, which is not achieved.<br><br>The cumulative Z-curve does not cross the conventional (straight dark red line) and TSA-adjusted boundaries (bright red line) for benefit, showing no statistical or clinical benefit in reducing acute kidney injury.
Mechanical ventilation time	Trial sequential analysis for mortality between both groups of patients. The required information size is 241, which is achieved.

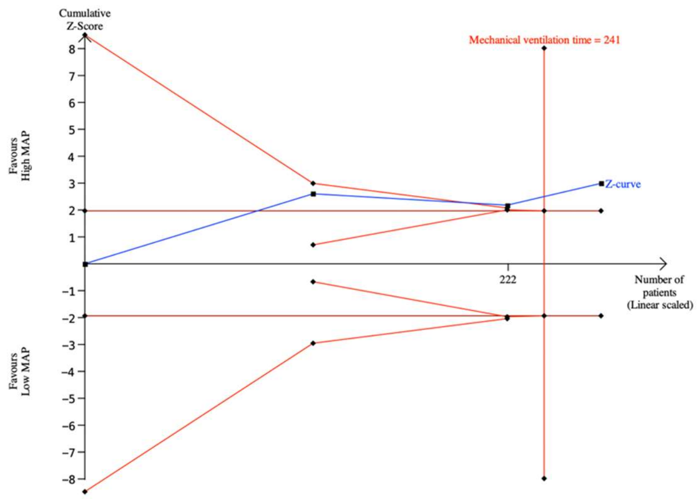

The cumulative Z-curve also crosses both the conventional (straight dark red line) and TSA-adjusted boundaries (bright red line) for benefit, showing clinical benefit in reducing mortality.

ICU length of stay

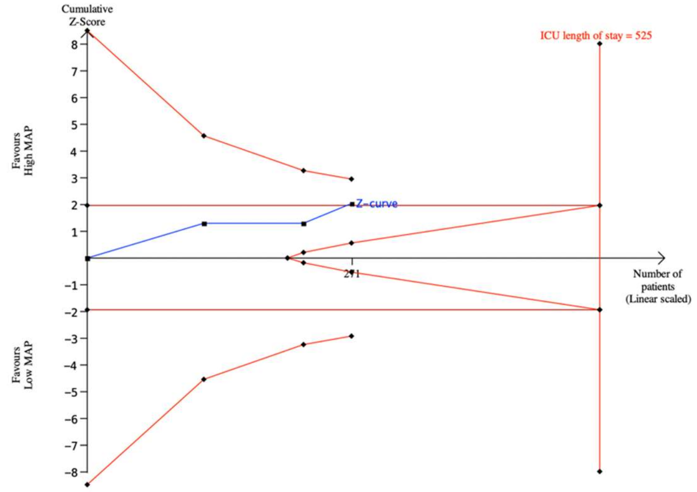

Trial sequential analysis for mortality between both groups of patients. The required information size is 525, which is not achieved.

The cumulative Z-curve crosses the conventional (straight dark red line) but not TSA-adjusted boundaries (bright red line) for benefit, showing statistical benefit in reducing mortality, but this has yet to translate to meaningful clinical benefit.

Supplementary Table S11: Forest plots for other secondary outcomes

Outcome: Levels of neuron-specific enolase/ mcg/L

Forest plot:

Study	High MAP Total	High MAP Mean	High MAP SD	Low MAP Total	Low MAP Mean	Low MAP SD	MD	95%-CI	Weight
Ameloot 2019	51	58.00	51.87	51	63.67	74.00	-5.67	[-30.47; 19.13]	0.8%
Grand 2020	23	25.00	24.50	26	21.33	14.12	3.67	[-7.72; 15.06]	3.8%
Jakkula 2018	60	22.17	13.14	60	23.73	15.04	-1.56	[-6.61; 3.49]	19.4%
Kjaergaard 2022	393	22.00	19.35	396	21.00	17.11	1.00	[-1.55; 3.55]	76.0%
**Random effects model**	**527**			**533**			**0.55**	**[-1.67; 2.78]**	**100.0%**

Heterogeneity: $I^2 = 0\%$, $\tau^2 = 0$, $p = 0.72$

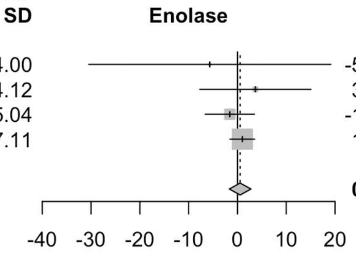

Outcome: Incidence of arrhythmias

Forest plot:

Study	High MAP Events	High MAP Total	Low MAP Events	Low MAP Total	OR	95%-CI	Weight
Ameloot 2019	7	51	17	51	0.32	[0.12; 0.85]	44.2%
Kjaergaard 2022	59	393	50	396	1.22	[0.81; 1.83]	55.8%
**Random effects model**		**444**		**447**	**0.67**	**[0.18; 2.50]**	**100.0%**

Heterogeneity: $I^2 = 84\%$, $\tau^2 = 0.7586$, $p = 0.01$

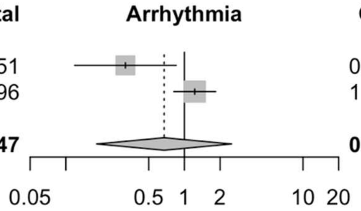

Outcome: Incidence of acute kidney injuries

Forest plot:

Study	High MAP Events	High MAP Total	Low MAP Events	Low MAP Total	OR	95%-CI	Weight
Grand 2020	3	23	8	26	0.34	[0.08; 1.47]	29.8%
Kjaergaard 2022	41	393	40	396	1.04	[0.65; 1.64]	70.2%
**Random effects model**		**416**		**422**	**0.74**	**[0.27; 2.03]**	**100.0%**

Heterogeneity: $I^2 = 51\%$, $\tau^2 = 0.3208$, $p = 0.15$

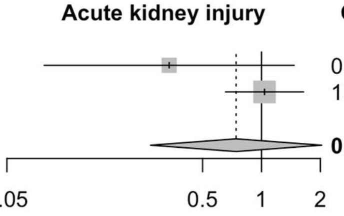

Duration of mechanical ventilation/ days

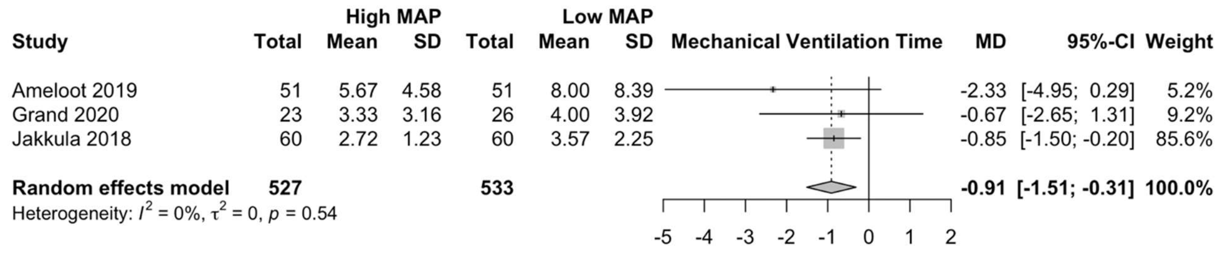

Duration of intensive care unit stay/ days

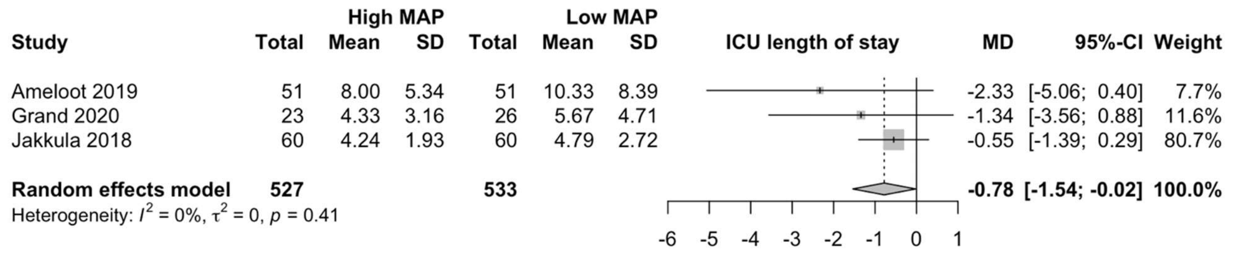

Supplementary Table S12a: Raw data for outcomes in included trials

Study	MAP group/ mmHg*	Mortality	Favourable neurological outcome**	Arrhythmia	NSE levels mcg/L	Other complications
Ameloot 2019	Low: 65	29/51	18/51	17/51	Day 0: 38 (28-46)<br>Day 1: 42 (28-68)<br>Day 2: 42 (26-123)<br>Day 3: 30 (18-65)<br>Day 4: 20 (15-43)	% voxels with ADC score <650.10-6 mm2/s<br>Observed: 11 (8-15)<br>After imputation: 12 (9-16)<br>After imputation with non-survivors set to 100%: 16 (12-21
	High: 85 to 100	30/51	21/51	7/51	Day 0: 39 (29-46)<br>Day 1: 46 (34-67)<br>Day 2: 42 (32-100)<br>Day 3: 41 (25-170)<br>Day 4: 31 (21-97)	% voxels with ADC score <650.10-6 mm2/s<br>Observed: 11 (8-18)<br>After imputation: 16 (13-21)<br>After imputation with non-survivors set to 100%: 21 (15-28)
Grand 2020	Low: 65	9/26	13/26		24 hours: 28 (20-41)<br>48 hours: 20 (13-31)<br>Peak: 29 (21-42)	ICU length of stay/days: 5 (3-9)<br>Hospital length of stay/days: 13 (8-29)<br>Duration of mechanical ventilation/days: 3 (2-7)<br>Duration of vasopressor use/days: 2 (2-3)<br>RRT: 8<br>Severe bleeding during admission: 2
	High: 72	10/23	13/23		24 hours: 21 (18-30)<br>48 hours: 18 (13-44)<br>Peak: 23 (19-44)	ICU length of stay/days: 5 (2-6)<br>Hospital length of stay/days: 9 (7-14)<br>Duration of mechanical ventilation/days: 4 (1-5)<br>Duration of vasopressor use/days: 2 (1-4)<br>RRT: 3<br>Severe bleeding during admission: 1
Jakkula 2018	Low: 65 to 75	20/60	37/60		21.2 (15.1-34.9)	Mechanical ventilation duration/hours: 82 (52-123)
	High: 80 to 100	18/60	41/60		22 (13.6-30.9)	Mechanical ventilation duration/hours: 59 (49-88)

Kjaergaard	Low: 63	114/396	269/396	50/396	18 (11-34)	Seizure: 88<br>Infection: 110<br>Any bleeding: 92<br>Uncontrolled bleeding: 16<br>Electrolyte disorder: 34<br>Metabolic disorder: 31
	High: 77	122/393	260/393	59/393	18 (11-37)	Seizure: 76<br>Infection: 102<br>Any bleeding: 82<br>Uncontrolled bleeding: 22<br>Electrolyte disorder: 23<br>Metabolic disorder: 31

Supplementary Table S12b: Raw data for outcomes in observational studies in sensitivity analysis

Study	MAP group/ mmHg*	Mortality	Favourable neurological outcome**	Arrhythmia	NSE levels mcg/L	Other complications
Grand 2019	Low: <70	86/188		53/188		AKI: 102<br>AKI: 25
	High: 70 to 80 (75)	162/364		103/364		AKI: 164<br>RRT: 43
	High: >80	137/299		74/299		AKI: 126<br>RRT: 13
Russo 2017	Low: <70	11/20	8/20			Major bleeding: 10
	High: 70 to <80	14/67	49/67			Major bleeding: 25
	High: ≥ 80	4/35	26/35			Major bleeding: 13

Note:
*: If an MAP range was given, MAP was taken as the midpoint of the range
**: Favourable neurological outcome was defined as a Cerebral Performance Category of 1 or 2, or a modified Rankin score of 0 to 2
MAP, mean arterial pressure; NSE, neutron-specific enolase; ADC, apparent diffusion coefficient; ICU, intensive care unit; RRT, renal replacement therapy; AKI, acute kidney injury
